# Human Rabies Survivors in India: An Emerging Paradox?

**DOI:** 10.1371/journal.pntd.0004774

**Published:** 2016-07-28

**Authors:** Reeta Subramaniam (Mani)

**Affiliations:** Department of Neurovirology, WHO Collaborating Centre for Reference and Research on Rabies, National Institute of Mental Health and Neurosciences (NIMHANS), Bangalore, Karnataka, India; Wistar Institute, UNITED STATES

On a particularly hot afternoon early last year, a couple—barefoot and in tattered clothes—walked into the emergency services at the National Institute of Mental Health and Neurosciences (NIMHANS), a public neurocare hospital in Bangalore, India. The woman held a sick child in her arms and her male companion followed with two more children in tow.

The child was diagnosed to have rabies encephalitis—apparently acquired through a stray dog bite a month prior, for which medical care was not sought. The grim prognosis was conveyed to the parents, who were migrant labourers in the city. The child died within two hours of admission. However, there was nobody to claim the mortal remains of the young child; the parents had discreetly left the hospital premises and they could not be traced. The body lay in the mortuary, unclaimed for several weeks before it was cremated by the police.

*The cost of cremating a dead child could be better utilized to feed other hungry mouths*—this is the harsh reality faced by many mothers in India, their grief numbed by poverty.

Rabies, a fatal disease, yet almost 100% preventable by timely and appropriate postexposure prophylaxis (PEP), continues to kill about 20,000 people every year in India, accounting for almost a third of the 61,000 global human deaths due to rabies [1,2]. This figure may be an underestimate because rabies is not a notifiable disease in India, and systematic surveillance for animal and human rabies is not done.

These tragic deaths continue to happen primarily because a majority of victims do not receive rabies vaccination, and a few of those who do do not complete the full course. Moreover, the use of rabies immunoglobulins (RIG)is abysmally low [2]. This serious lapse in PEP can be attributed to the lack of awareness about the potential seriousness of animal bites and the need for prompt PEP in the community as well as among medical professionals and an irregular supply of antirabies vaccines and RIG, particularly in primary health care facilities. In addition, some dog bite victims cannot afford the cost of PEP or may resort to indigenous treatment practices [2].

At the other end of the dismal spectrum of the rabies scenario in India, an incredibly paradoxical finding has emerged in the recent past. Survival from human rabies (albeit with severe residual deficits) has been reported in 6 patients in the last 6 years from India [3–8], almost unheard of until 2010 except for a single case of partial recovery from rabies reported in 2002 [9]. It is a well-known fact that survival from rabies is extremely rare, and only about 15 human survivors from rabies have been reported globally [3,6,8]. In fact, we imbibed the tenets of rabies diagnosis as students of medicine decades ago: “If a patient has rabies, he will die in the next few days; if he does not die, he does not have rabies!”

The Neurovirology laboratory at NIMHANS, Bangalore, a WHO Collaborating Centre for Reference and Research on rabies, receives samples from clinically suspected human rabies cases all over India for diagnostic confirmation. In addition to the 6 reported survivors since 2010, over a period of 3 years (2012–2014), 6 patients have been reported to have had prolonged survival of 2 weeks to 3 months after onset of illness [8], unusual in victims of rabies, for whom the average interval from clinical disease onset to death has been reported to be 5.7 days in furious rabies and 11 days in paralytic rabies [10]. At least a few more, if not many such cases, may be unreported from various hospitals in India, probably for want of a laboratory confirmation of rabies, which remains a challenge [8].

What could possibly explain the recent unusual surge of human rabies survivors in India?

While public health facilities—the only choice for the socioeconomically underprivileged population in India—may be found wanting, especially in rural areas, in a stark contradiction, several private and some public medical institutes offering world-class medical care have been recognized in India in the last decade or so. Besides being a hub for medical tourism, these advanced medical facilities are accessible to a considerable segment of the Indian population who are economically secure and are covered by health insurance. Access to excellent intensive care facilities and an aggressive approach of management with supportive care may be one of the most important factors contributing to prolonged survival of human rabies cases in India in the recent past. Some of these patients had atypical clinical manifestations, and availability of antemortem diagnostic facilities and the treating physicians’ efforts to obtain an intravitam laboratory confirmation could also have played a critical role. It is likely that the well-publicized survival and almost complete recovery of a teenager in the United States using the “Milwaukee protocol” in 2004 after she developed rabies [11] may have provided an impetus for physicians to attempt aggressive management of human rabies.

Sadly, “survival” has not always been synonymous with “recovery,” and all except one of the reported human survivors in India are left with poor functional outcomes—a tragic occurrence with serious long-term repercussions for family members. Physicians and caregivers are therefore faced with the agonizing dilemma of whether “to treat or not to treat” patients with a diagnosis of rabies. Most public hospitals cannot justify expenditure from already limited resources on a patient with rabies who has statistically negligible chances of complete recovery, leading to several medical, ethical, legal, social, and economic challenges.

Current medical outcomes, however, do not predict future medical outcomes, as is amply evident by the commendable strides in medicine over the last few decades. The dismal functional outcomes in most Indian patients with rabies should not paralyze progress in the pursuit of saving precious human lives. Moreover, almost complete recovery has also been reported in a few rabies survivors from India and elsewhere [3,7,11].

Currently, most patients with a suspected diagnosis of rabies are referred to state-run “isolation hospitals,” which can offer not much except a dignified death in the form of an earmarked hospital bed. Incremental triage of potential candidates for aggressive management and their referral to tertiary public hospitals with advanced medical facilities should be practiced in resource-limited settings in India to focus efforts on subjects that are most likely to be survivors—young, immunocompetent individuals with prior vaccination, early appearance of rabies, neutralising antibodies in CSF and serum, and mild neurological illness at initiation of therapy [12]. Additionally, medical institutes in India engaged in management of human rabies should encourage clinical trials with newer, promising antivirals and/or biologicals and motivate researchers to explore newer therapeutic strategies—a ray of hope for unfortunate victims of this virtually fatal disease. A few public and also private medical academic institutes in India will be certainly prepared to take on this challenge.

More importantly however, the focus on treatment and management of rabies should not draw away attention from the core objective, which is unarguably the “prevention” of human rabies.

Routine pre-exposure immunization of at least the most vulnerable population—children—should be considered, though it may sound preposterous in a country where most human rabies deaths are known to occur because of lack of PEP.

There is an acute shortage of rabies biologicals, especially RIG, reported from time to time from several states in India. The WHO also reports a critical shortage of RIG worldwide. This is because both equine and human RIG can be manufactured only in limited quantities for several reasons. In India, only equine RIG is indigenously manufactured because of high production costs for human RIG. In the recent past, various studies and clinical trials have reported the production and evaluation of human monoclonal antibodies that are equally or more potent than RIG and have been found to be promising substitutes that can bring down the cost of PEP considerably [13,14,15]. One of these products [15], manufactured by Serum Institute of India, is set to be launched this year and will hopefully resolve the RIG crisis in India, at least to some extent.

To address the shortage of vaccines, India needs to scale up indigenous production of modern cell culture vaccines. More importantly, intradermal vaccination, which brings down the cost of PEP significantly, should be expanded to more areas across various states. This can be achieved by training of medical and nursing staff in this technique. The Global Alliance for Vaccines and Immunization (GAVI) does not currently support funding for rabies vaccines or immunoglobulins; however, recently it has decided to invest in research on feasibility of GAVI support for rabies vaccines.

Rabies deaths are scattered and, sadly, never manage to garner the critical attention that an epidemic or outbreak can achieve, which is one of the reasons why rabies continues to be a neglected disease in India despite continuing to cause significant human mortality. Recently, however, the Ministry of Health and Family Welfare, Government of India has initiated the National Rabies Control Programme under the 12th 5-year plan, which has both animal and human components. Increasing awareness of rabies and PEP among the public and health care professionals should be foremost on their agenda to prevent tragic human deaths.
