# Correction: Human Rabies Survivors in India: An Emerging Paradox?

**DOI:** 10.1371/journal.pntd.0004975

**Published:** 2016-08-24

**Authors:** Reeta Subramaniam Mani

The first author's name is spelled incorrectly. The correct name is: Reeta Subramaniam Mani. The correct citation is Mani RS (2016) Human Rabies Survivors in India: An Emerging Paradox? PLoS Negl Trop Dis 10(7): e0004774. doi: 10.1371/journal.pntd.0004774
